# Mitochondrial CLPP in Health and Disease: Mechanisms, Therapeutic Duality and Emerging Opportunities

**DOI:** 10.1002/jimd.70247

**Published:** 2026-09-03

**Authors:** Lea Isermann, Aleksandra Trifunovic

**Affiliations:** ^1^ Faculty of Medicine, University Hospital Cologne, Institute for Mitochondrial Diseases in Ageing and Cologne Excellence Cluster on Cellular Stress Responses in Ageing‐Associated Diseases (CECAD), University of Cologne Cologne Germany; ^2^ Center for Molecular Medicine Cologne (CMMC), University of Cologne Cologne Germany

## Abstract

Mitochondrial CLPP has emerged as an unusual therapeutic target because both increasing and decreasing its proteolytic activity can be beneficial, depending on the cellular and disease context. Pharmacological CLPP hyperactivation drives broad degradation of mitochondrial proteins and can selectively collapse mitochondrial fitness in susceptible tumor cells, an approach now clinically validated by the approval of dordaviprone for mutant diffuse midline glioma. Conversely, reduced CLPP activity can preserve respiratory‐chain components and promote adaptive metabolic and redox remodelling in selected models of mitochondrial disease, neurodegeneration and metabolic dysfunction, with emerging potential in ischaemia‐reperfusion injury. These opposing outcomes reflect the broader role of CLPXP in controlling mitochondrial translation, respiratory‐chain integrity and metabolism rather than acting simply as a general protein quality‐control system. In this review, we discuss the physiological functions and substrate selectivity of CLPXP, the mechanistic basis and clinical development of CLPP inhibitors and activators, and the growing evidence that therapeutic responses depend strongly on tissue identity, metabolic state and the nature of the underlying mitochondrial defect. Together, these findings position CLPP as a context‐dependent therapeutic switch whose activity may need to be tuned in opposite directions to either preserve mitochondrial resilience or selectively dismantle mitochondrial fitness.

## Graphical abstract

The graphical abstract illustrates the context‐dependent therapeutic modulation of CLPP. Pharmacological CLPP activation has reached clinical application in cancer (black arrow), whereas reduced CLPP activity has shown beneficial effects experimentally in models of primary mitochondrial disease and metabolic syndrome, as well as cancer (grey arrows).

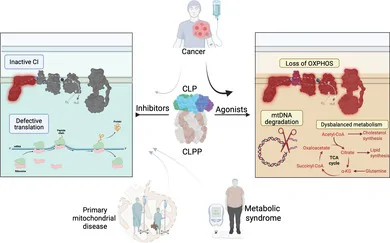

## 1 Introduction

Mitochondrial protein homeostasis depends on more than the removal of damaged proteins. Because the mitochondrial proteome is assembled from proteins encoded by both mitochondrial and nuclear genomes, its composition must be continuously coordinated with organelle metabolism, gene expression and respiratory‐chain activity [1]. Mitochondrial chaperones and proteases are central to this regulation, acting not only as quality‐control factors but also as selective modulators of mitochondrial function [2, 3].

The ATP‐dependent CLPXP protease exemplifies this broader regulatory role. It is composed of the AAA+ unfoldase CLPX, which recognizes and translocates substrates, and the serine protease CLPP, which degrades them within an enclosed proteolytic chamber [4, 5]. Rather than mediating indiscriminate clearance of damaged proteins [6], CLPXP controls the abundance and turnover of selected components of the mitochondrial translation machinery [7], respiratory chain [8, 9], and metabolic pathways [10, 11, 12, 13, 14]. Through these substrates, it influences oxidative phosphorylation, redox balance, and cellular adaptation to mitochondrial stress [5].

These functions have placed CLPP at the centre of an emerging therapeutic paradox [15]. Pharmacological hyperactivation can drive uncontrolled mitochondrial proteolysis and bioenergetic collapse in tumor cells [8], whereas reduced CLPP activity may preserve respiratory‐chain components or induce protective metabolic remodelling in selected non‐malignant disorders [9, 16]. This review examines the mechanistic basis of this bidirectional vulnerability and evaluates the therapeutic potential and limitations of targeting CLPP in cancer, mitochondrial disease, metabolic dysfunction and ischaemic injury.

### 1.1 Molecular Architecture and Physiological Functions of CLPXP

CLPP is a nuclear‐encoded mitochondrial serine protease that is evolutionarily conserved from bacteria to humans. The mature human CLPP predominantly forms an asymmetric heptameric ring [17]. Upon engagement by hexameric CLPX, a second CLPP ring is recruited to generate the tetradecameric proteolytic barrel of approximately 340 kDa that is assembled into the CLPXP complex [4]. The catalytic sites, each containing a canonical Ser‐His‐Asp triad, face the interior of this chamber [4, 18]. Narrow, gated pores at both ends of the barrel restrict access to the active sites, limiting the intrinsic activity of CLPP alone largely to short, unstructured peptides [19]. Processive degradation of folded proteins requires association with the CLPX unfoldase, which uses cycles of ATP binding and hydrolysis to recognize, unfold, and translocate substrates through its central pore into the CLPP chamber [4]. CLPX assembles as a homohexameric ring and docks on one or both apical surfaces of CLPP through conserved LGF‐loop motifs, which insert into hydrophobic pockets formed between adjacent CLPP subunits [17]. Human CLPX additionally contains a eukaryote‐specific E‐loop that stabilizes hexamer formation and supports ATPase activity [17]. Peptide binding within the CLPP active sites induces further structural rearrangements that convert the assembled complex into a fully proteolytically competent state [17]. Together, these findings support a model in which human CLPXP is activated through sequential CLPX engagement, CLPP double‐ring assembly, and substrate‐induced maturation of the proteolytic chamber [17].

The evolutionary conservation of CLPXP from bacteria to mammals underscores its fundamental role in cellular and mitochondrial protein homeostasis [20]. The physiological importance of CLPP is further illustrated by the pronounced consequences of its loss across species [20].

In humans, biallelic CLPP variants cause Perrault syndrome Type 3, an autosomal‐recessive disorder characterized predominantly by sensorineural hearing loss and ovarian dysfunction ranging from ovarian dysgenesis to premature ovarian insufficiency [21, 22, 23, 24, 25]. Neurological manifestations, including learning difficulties, epilepsy, dystonia, and spastic‐ataxic features, occur in a substantial subset of patients [26]. Recent genotype–phenotype analyses suggest that neurological involvement is more frequent in individuals carrying biallelic truncating variants or combined truncating and missense variants than in those with biallelic missense variants, indicating that the degree of residual CLPP function may contribute to disease severity [24, 27].

CLPP‐deficient mice recapitulate major features of the human disease, including infertility, progressive hearing loss, growth retardation and sub‐Mendelian birth ratios, yet surviving animals can maintain a largely normal lifespan [10, 28]. At the mitochondrial level, CLPP loss causes defects in mitoribosome assembly and mitochondrial translation, progressive respiratory‐chain dysfunction, and impaired turnover of vulnerable complex I (CI) subunits [5, 7, 10, 28]. Remarkably, however, its metabolic consequences are not uniformly detrimental: CLPP‐deficient mice show improved glucose homeostasis and resistance to diet‐induced obesity, while exhibiting defective adaptive thermogenesis and reduced metabolic flexibility [10, 11].

Despite the broad consequences of CLPP deficiency, only a limited number of mammalian proteins are firmly established as physiological CLPXP substrates. Unlike LONP1, CLPXP appears to act preferentially on a defined set of regulatory or functionally vulnerable proteins rather than serving as a general disposal system for the mitochondrial matrix proteome [6, 7, 29]. Recent work suggests that serine phosphorylation may facilitate substrate selection by promoting recognition by the RKL loop of CLPX and enhancing CLPXP‐dependent degradation in biochemical and cellular assays [30]. This provides a potential mechanism by which specific mitochondrial proteins are marked for regulated turnover, although the responsible kinases, the prevalence of this signal among endogenous substrates and its physiological regulation remain to be established.

ERAL1 is the best‐characterized regulatory substrate. This 12S rRNA chaperone accumulates and remains abnormally associated with the small mitoribosomal subunit in CLPP‐deficient mitochondria [7]. Its normalization partially restores mitoribosome assembly, mitochondrial translation and respiratory‐chain biogenesis, supporting a model in which timely CLPXP‐mediated removal of ERAL1 enables maturation of the functional 55S ribosome [7]. EFG1, a mitochondrial translation elongation factor, also accumulates and is captured by inactive CLPP but remains less rigorously validated.

Respiratory chain proteins constitute an important class of CLPXP substrates. Within CI, CLPXP selectively turns over the matrix‐exposed N‐module, enabling a dedicated salvage pathway in which damaged or expended components are removed and replaced without disassembling the entire complex [7, 9, 14, 31]. This preserves CI function at a substantially lower energetic cost than complete *de novo* reassembly [9]. Beyond CI, the complex II (CII) subunits SDHA and SDHB undergo CLPXP‐dependent degradation in several cellular models [7, 8, 32]. In acute myeloid leukaemia cells, CLPXP limits the accumulation of dysfunctional SDHA, thereby maintaining CII activity and supporting proliferation [32]. UQCRC1, a matrix‐exposed component of complex III (CIII), has also been identified as a CLPXP substrate and appears to undergo stepwise proteolysis [14]. Proteomic screens have further identified subunits of complex V (CV) and other respiratory‐chain proteins as potential CLPXP targets, although most still lack direct biochemical validation [7]. Similarly, other proposed substrates include NOA1, ALAS1 and proteins involved in amino‐acid, fatty‐acid and tricarboxylic‐acid metabolism, but most remain candidates rather than conclusively demonstrated direct targets [7, 10, 14, 31, 33]. Proteins degraded after pharmacological CLPP hyperactivation should also be distinguished from physiological substrates, because CLPP agonists bypass normal CLPX‐dependent substrate selection and induce broad proteolysis in the mitochondrial matrix.

### 1.2 Therapeutic Targeting of CLPP in Cancer

CLPP has emerged as an unusual anticancer target because tumor growth can be impaired by either inhibiting or hyperactivating its proteolytic activity [15, 32, 34] Inhibition disrupts mitochondrial protein quality control [32], whereas pharmacological activation uncouples proteolysis from normal substrate recognition and drives excessive degradation of mitochondrial proteins [8, 35]. Although mechanistically opposite, both interventions can collapse respiratory‐chain function and mitochondrial metabolism [8, 32]. Their efficacy, however, is strongly dependent on tumor lineage, metabolic state and reliance on oxidative phosphorylation.

Altered CLPP expression has been reported in haematological malignancies and several solid tumors, but expression is heterogeneous and does not uniformly predict therapeutic response [15, 36]. Tumors with high mitochondrial activity, increased proteotoxic stress or limited metabolic flexibility may become particularly dependent on CLPP‐mediated proteostasis.

The strongest evidence comes from acute myeloid leukaemia (AML), where CLPP protein is elevated in approximately 45% of primary samples across diverse molecular subtypes [8, 32, 37]. Genetic depletion or pharmacological inhibition preferentially reduces the viability of CLPP‐high AML cells, while normal haematopoietic progenitors are comparatively resistant [8, 32, 37]. CLPP expression has also been linked to tumor behavior in breast, ovarian, pancreatic, hepatic, colorectal, and lung cancer, although the direction and prognostic significance vary between tumor types [36, 38]. Thus, CLPP should be viewed as a context‐dependent mitochondrial dependency rather than a universal oncogenic factor.

#### 1.2.1 Mechanisms of CLPP Inhibition and Hyperactivation

CLPP inhibitors block normal substrate turnover, leading to the accumulation of dysfunctional or improperly assembled proteins. In cancer cells with a high mitochondrial proteostasis burden, this can impair oxidative phosphorylation, increase mitochondrial stress and trigger apoptosis. The therapeutic rationale for CLPP inhibition is best established in AML [32, 39]. CLPP depletion or chemical inhibition reduces respiratory activity, promotes cytochrome c release and activates intrinsic apoptosis in susceptible leukaemia cells.

Several experimental inhibitors have been developed, including the β‐lactone A2‐32‐01 and phenyl ester compounds such as TG42 and TG53 [32, 40, 41] (Table 1). These compounds inhibit the catalytic serine of CLPP and show antiproliferative activity in cancer cells. However, their broader reactivity with other serine hydrolases remains a major limitation [15]. Current inhibitors therefore serve mainly as experimental tools, and clinical development will require greater selectivity, improved mitochondrial delivery and pharmacodynamic markers that demonstrate target engagement (Table 1). Potential biomarkers for inhibitor sensitivity include high CLPP abundance, strong dependence on oxidative phosphorylation and accumulation of validated CLPP substrates following treatment [15]. Chronic toxicity must also be considered because sustained CLPP deficiency impairs mitochondrial function in several metabolically active tissues [7, 10].

**TABLE 1:** List of human and bacterial CLPP targeting compounds including activators, inhibitors, and other deregulators.

Compound	Class	Mechanism	Target	Disease area/application	Development status	References
Human mitochondrial CLPP activators						
Dordaviprone (ONC201, Modeyso)	Imipridone	Allosteric CLPP hyperactivator + DRD2 antagonist	Human mitochondrial CLPP	H3 K27M‐mutant diffuse midline glioma	FDA‐approved (accelerated approval, Aug 2025)	US Food & Drug [42]
Dordaviprone/ONC201	Imipridone	CLPP agonist	Human mitochondrial CLPP	Pan‐cancer (mechanistic study)	Mechanistic discovery paper	Graves et al. [35]
JZP3507/ONC206	Imipridone (ONC201 analogue) (Jazz Pharmaceuticals)	CLPP agonist	Human mitochondrial CLPP	Diffuse Midline Glioma (DMG)/malignant solid tumors incl. GLIOBLASTOMA	Preclinical/Early clinical studies	Agren and Sahlholm [43]
ONC212	Imipridone (ONC201 analogue)	CLPP agonist	Human mitochondrial CLPP	Breast cancer, pancreatic cancer (preclinical)	Preclinical/Early clinical studies	Xu et al. [44]
IMP075	Imipridone (ONC201 analogue, optimized)	CLPP agonist	Human mitochondrial CLPP	Colorectal cancer	Preclinical	Zhang et al. [45]
NCA029	ClpP agonist derived from CCG1423 (non‐imipridone scaffold)	CLPP agonist	Human mitochondrial CLPP	Colorectal cancer; also explored in inflammatory bowel disease	Preclinical	Zhang et al. [46]
ONC213	Imipridone (ONC201 analogue)	CLPP agonist	Human mitochondrial CLPP	Glioma (preclinical)	Preclinical	Daglish et al. [47]
TR‐57	“TR” compound (ClpP‐optimized, non‐imipridone scaffold) (Madera Therapeutics)	CLPP agonist	Human mitochondrial CLPP	Triple‐negative breast cancer (preclinical)	Preclinical	Fatima et al. [48]
TR‐107	“TR” compound (ClpP‐optimized, non‐imipridone scaffold) (Madera Therapeutics)	CLPP agonist	Human mitochondrial CLPP	Triple‐negative breast cancer, diffuse midline glioma (preclinical)	Preclinical	Fennell et al. [49]
Compound 9 (MS6076)	Next‐generation ClpP agonist	CLPP agonist	Human mitochondrial CLPP	Breast cancer (preclinical)	Preclinical	Xu [44]
ZG36	Diacylpyrimidine (ICG‐001/ZG111‐derived scaffold)	CLPP agonist	Human mitochondrial CLPP	Acute myeloid leukemia	Preclinical	Zhang et al. [50]
ZK53	Simple non‐rigid scaffold (distinct from ADEPs/imipridones)	CLPP agonist	Human mitochondrial CLPP selective (no effect on bacterial ClpP)	NSCLC/lung squamous cell carcinoma; also explored in SCLC ferroptosis sensitization	Preclinical	Zhou et al. [51]
7 k	Ring‐opened imipridone‐derived scaffold	CLPP agonist	Human mitochondrial CLPP	Acute myeloid leukemia	Preclinical	Xiang et al. [52].
CLPP‐1071	Optimized non‐imipridone activator scaffold	CLPP agonist	Human mitochondrial CLPP	Not yet studied for tumor‐type specificity	Preclinical	Chen et al. [53]
THX6	Tetrahydropyrido[4,3‐d]pyrimidine‐2,4(1H,3H)‐dione scaffold	CLPP agonist	Human mitochondrial CLPP	Not yet studied for broad tumor‐type specificity; characterized in DIPG/DMG brain tumor models	Preclinical	Miciaccia et al. [54]
ADEP‐41 (human/mitochondrial ClpP activation)	Cyclic acyldepsipeptide (ADEP analogue)	Developed against Bacterial ClpP. Activates human mitochondrial CLPP at the same H‐pockets used in bacteria, displacing CLPX	Human mitochondrial CLPP	Pan‐cancer cell line panel (HEK293, HeLa, U2OS, SH‐SY5Y)	Preclinical	Wong et al. [55]
Human mitochondrial CLPP inhibitors						
A2‐32‐01	β‐lactone (oxetanone)	Covalent active‐site Ser inhibitor	Human mitochondrial CLPP	Acute myeloid leukemia (research)	Research tool	Cole et al. [32]
(3R,4R)‐A2‐32‐01	β‐lactone enantiomer	Covalent CLPP inhibitor	Human/ *S. aureus* CLPP inhibitor	Chemical biology (research)	Research tool	Gersch et al. [56]
AV167	Phenyl ester (from the same screen as ML90)	Covalent acyl‐enzyme trap (active‐site Ser)	Human mitochondrial CLPP	Chemical biology (research)	Research tool	Hackl et al. [41]
TG42/TG53 (human‐selective AV167 analogues)	Phenyl ester (naphthofuran‐substituted AV167 analogues)	Covalent active‐site Ser inhibitor, preferentially of human over bacterial ClpP	Human mitochondrial CLPP	Hepatocellular carcinoma (Huh7 cells)	Research tool	Gronauer et al. [40]
α‐Aminoboronic acids (8a, 8b, 8c)	Peptide boronate	Reversible covalent active‐site binding (proposed interaction with Ser97/His122)	Human mitochondrial CLPP	Chemical biology (research)	Research tool	Tan et al. [57]
Bacterial ClpP activators/Deregulators						
ADEP1–ADEP4 and synthetic analogues	Cyclic acyldepsipeptide (ADEP)	Forces open the ClpP axial pore → unregulated, ATPase‐independent proteolysis	Bacterial ClpP	Gram‐positive infections (MRSA, VRE, drug‐resistant pneumococcus, persister/biofilm infections)	Preclinical/extensively optimized research series	Brötz‐Oesterhelt et al. [58]
ADEP mechanistic probes (conformational‐control study)	Cyclic acyldepsipeptide + chemical probes	Allosteric conformational activation of ClpP (beyond simple pore‐opening)	Bacterial ClpP	Mechanistic study informing antibacterial ADEP design	Research (mechanistic)	Gersch et al. [59]
ADEP4 (FtsZ‐degradation mechanism)	Cyclic acyldepsipeptide (ADEP)	ADEP‐activated ClpP degrades the cell‐division protein FtsZ	Bacterial ClpP	Antibacterial/antibiofilm mechanism	Mechanistic study (Brötz‐Oesterhelt lab)	Sass et al. [60]
ADEP analogues (conformationally restricted)	Cyclic acyldepsipeptide	ClpP pore activation (optimized binding/stability)	Bacterial ClpP	Gram‐positive infections ( *S. aureus* , *E. faecalis* , *S. pneumoniae* )	Medicinal chemistry/preclinical	Carney et al. [61]
ACP1 series (activators of self‐compartmentalized proteases 1)	Small molecule (non‐ADEP scaffold)	ClpP pore activation via electrostatic network reorganization	Bacterial ClpP	Antibacterial ( *N. meningitidis* , *E. coli* ) — research	Research tool	Mabanglo et al. [62]
Bacterial ClpP inhibitors						
β‐lactones (ClpP1P2‐active series)	β‐lactone	Covalent modification of ClpP2 active‐site Ser	*M. tuberculosis* /actinobacterial ClpP1P2	Tuberculosis (early discovery)	Research/early discovery	Compton et al. [63]
GDI‐5755	Phenyl ester	Covalent ClpP1 active‐site Ser inhibitor	*M. tuberculosis* ClpP1P2	Tuberculosis	Research/lead optimization	Zhang et al. [50]
β‐lactones (original anti‐virulence series)	β‐lactone	Covalent active‐site Ser inhibitor (attenuates virulence at sub‐lethal doses)	*S. aureus* (incl. MRSA) ClpP	Antivirulence strategy for *S. aureus* infection (research)	Research/discovery	Böttcher and Sieber [64]
Second‐generation β‐lactones	β‐lactone (optimized series)	Covalent active‐site Ser inhibitor	*S. aureus* ClpP	Antivirulence strategy (research)	Research/medicinal chemistry	Zeiler et al. [65]
β‐sultams (RKS07)	β‐sultam (cyclic sulfonamide)	Sulfonylates active‐site Ser98, inducing dehydroalanine formation and disassembly of the tetradecamer into inactive heptamers	Bacterial ClpP	Chemical biology (research)	Research tool	Gersch et al. [66]
Tailored phenyl esters (MAS series)	Phenyl ester (optimized substituents)	Covalent active‐site Ser inhibitor of ClpXP	*S. aureus* ClpP/ClpXP	Antivirulence strategy — attenuates alpha‐hemolysin secretion (research)	Research tool	Schwarz et al. [67]
Cystargolide A	Natural product (peptidic, β‐lactone‐related)	Covalent active‐site inhibitor	*S. aureus* ClpP	Structural/chemical biology (research)	Research tool	Illigmann et al. [68]
ML90 (phenyl ester series)	Phenyl ester	Covalent acyl‐enzyme trap (active‐site Ser)	Bacterial CLPP (selective; inactive on human ClpP)	Chemical biology (research)	Research tool	Hackl et al. [41]
Peptidomimetic boronate inhibitors	Boronic acid/boronate	Reversible covalent active‐site binding; stabilizes the ClpP tetradecamer	Bacterial ClpP	Chemical biology (research)	Research tool	Alves França et al. [69]
Mycobacterial boronate inhibitors (ClpP1P2)	Peptide boronate	Active‐site‐directed inhibitor of mycobacterial ClpP1P2	*M. tuberculosis* ClpP1P2	Tuberculosis (research)	Research tool	Akopian et al. [70]

CLPP agonists, exploit the same structural vulnerability from the opposite direction: instead of silencing CLPP, small molecules are used to constitutively activate it, causing it to activate proteolysis that bypasses normal CLPX‐dependent substrate selection and more broadly degrade substrates including essential respiratory chain subunits, mitochondrial translation factors and metabolic enzymes. They do so by binding to hydrophobic pockets on the apical surface of the protease, stabilizing an open‐pore conformation and activating degradation independently of normal CLPX‐mediated substrate selection [8, 35]. Frequently affected proteins include SDHA and SDHB, as well as components of CI, mitochondrial ribosomes and mtDNA‐maintenance machinery. Their degradation causes loss of respiratory capacity, membrane potential and ATP production, ultimately leading to proliferative arrest, senescence or apoptosis [8, 35]. CLPP hyperactivation also engages mitochondrial stress responses, frequently including the integrated stress response and ATF4‐dependent transcription that can contribute to growth arrest but may also provide partial adaptation, depending on tumor context [71]. Thus, insufficient and excessive proteolysis converge on a common endpoint: failure of mitochondrial metabolism.

The mechanistic principle of CLPP activators was established by acyldepsipeptides (ADEPs), natural product antibiotics that bind the same hydrophobic pockets and activate CLPP constitutively in bacteria [58] (Table 1). Subsequent work demonstrated that ADEP analogues similarly activate human CLPP, validating the hydrophobic pocket as a druggable site and providing the conceptual framework for the development of imipridones [55]. The bacterial compounds included in Table 1 are therefore not intended as therapeutic candidates for human disease, but illustrate the chemical and mechanistic foundations from which current strategies for pharmacological modulation of human mitochondrial CLPP have evolved.

The best‐characterized CLPP agonists are the imipridones, including ONC201, now known as dordaviprone, and the related compounds ONC206 and ONC212 [35] (Table 1). These molecules bind directly to CLPP and induce proteolysis independently of CLPX. Next‐generation imipridones are being developed to improve potency and central nervous system exposure. ONC206 is undergoing clinical evaluation, whereas ONC212 and several structurally distinct CLPP agonists remain preclinical. More potent activators have subsequently been developed, including TR‐series compounds and structurally distinct molecules such as ZG111 and ZK53 [34] (Table 1).

#### 1.2.2 Cancer‐Specific Vulnerabilities and Clinical Translation

AML is particularly sensitive to CLPP modulation [32]. Both inhibition and hyperactivation disrupt mitochondrial respiration and selectively kill subsets of AML cells [8, 32]. CLPP agonists can act independently of p53 and may therefore be effective across genetically diverse disease. Importantly, CLPP appears required for leukemic cell survival while remaining largely dispensable for normal hematopoietic cells, as CLPP loss in vivo does not impair normal hematopoiesis [8].

They may also be useful in combination therapies. AML stem and progenitor cells often depend strongly on oxidative phosphorylation, and mitochondrial metabolism contributes to resistance to the BCL‐2 inhibitor venetoclax. CLPP activation further weakens respiratory capacity and has shown cooperative activity with venetoclax in experimental AML and chronic lymphocytic leukaemia models [72].

Across solid tumors, CLPP hyperactivation has emerged as a treatment for mitochondrial vulnerability particularly in cancers with high respiratory activity, elevated proteotoxic stress or limited metabolic flexibility [15, 38].

*Pancreatic ductal adenocarcinoma* provides one of the strongest preclinical examples. CLPP agonists suppress tumor growth in cell‐line, xenograft and patient‐derived models, with ZG111 promoting respiratory‐chain degradation and mitochondrial collapse [34, 49, 73, 74, 75]. CLPX depletion or inhibition also disrupts mitochondrial proteostasis and increases stress and ferroptotic vulnerability in PDAC cells, although this is mechanistically distinct from CLPP activation [75]. Elevated CLPX expression has additionally been associated with poor prognosis.

In *hepatocellular carcinoma* and cancer stem‐cell models, CLPP agonists induce apoptosis through degradation of electron‐transport‐chain proteins, particularly SDHA and SDHB, whereas CLPP knockdown abolishes compound‐induced cytotoxicity [76].

In *breast cancer*, especially triple‐negative and HER2‐positive disease, ONC201, TR‐57 and related agonists suppress proliferation, mammosphere formation and tumor initiation by disrupting respiration and pathways required for stem‐cell maintenance, including mevalonate metabolism, MYC, HIF and YAP signalling [49, 77, 78, 79].

In *ovarian cancer*, CLPP activation reduces mitochondrial membrane potential, promotes cytochrome c release and induces apoptosis, with some studies suggesting that imipridones may overcome selected forms of chemoresistance [72, 80, 81, 82]. By contrast, CLPP activation alone has shown limited efficacy in *prostate cancer*, illustrating that respiratory disruption is not universally sufficient for tumor‐cell killing [83].

*Lung cancer* represents another relevant setting. In non‐small‐cell and lung squamous cell carcinoma, altered CLPP activity has been linked to metabolic reprogramming, DNA‐damage signalling and ferroptosis sensitivity [51, 84, 85, 86]. The agonist ZK53 causes CLPP‐dependent mitochondrial dysfunction and cell‐cycle arrest, and may increase ferroptotic vulnerability in selected models. Antitumour activity has also been reported in colorectal cancer and uveal melanoma, where high CLPP expression has been proposed as a potential marker of treatment sensitivity [45, 46, 87].

The most advanced clinical application of CLPP activation is in *H3 K27M‐mutant diffuse midline glioma*. Dordaviprone, formerly ONC201, penetrates the central nervous system and has produced durable responses in a subset of patients with recurrent disease [88, 89]. On 6 August 2025, the US Food and Drug Administration granted accelerated approval to dordaviprone for adult and paediatric patients aged 1 year or older with progressive H3 K27M‐mutant diffuse midline glioma following prior therapy [90]. This provided the first clinical validation of pharmacological activation of a mitochondrial protease as an anticancer strategy. Because approval was based on non‐randomized studies, confirmatory trials remain required.

The clinical success of Dordaviprone provides proof that CLPP activation can be therapeutically effective. However, sensitivity is strongly tumor‐specific; current inhibitors lack sufficient selectivity, and most agonists remain preclinical. Future work should define tumor‐specific substrate landscapes, establish robust metabolic biomarkers, and identify resistance mechanisms and rational drug combinations.

### 1.3 Reducing CLPP Activity in Mitochondrial Disease

Complete CLPP deficiency disrupts mitochondrial proteostasis and causes broad metabolic and respiratory remodelling, making it initially counterintuitive that reducing CLPP activity could ameliorate mitochondrial disease [7, 28]. Nevertheless, studies across several genetic models show that CLPP loss can become beneficial when the primary defect compromises mitochondrial translation or destabilizes the respiratory chain [16, 91].

This unexpected effect was first observed in mice lacking the mitochondrial aspartyl‐tRNA synthetase DARS2 in the heart. DARS2 deficiency severely disrupts mitochondrial translation, leading to OXPHOS failure, progressive cardiomyopathy and premature death [92]. Concomitant deletion of *Clpp* reduced cardiac hypertrophy and fibrosis, attenuated cytochrome c oxidase deficiency and extended median and maximal lifespan by approximately 35% [91]. The protective effect occurred without suppressing mitochondrial stress signalling, demonstrating that CLPP is not required for activation of the mammalian unfolded protein response (UPRmt). Instead, CLPP loss reduced the overall rate of mitochondrial translation while increasing productive synthesis of full‐length OXPHOS subunits and markedly decreasing the accumulation of abortive translation products [91]. Mechanistically, slower translation may prolong the availability of the limited Asp‐tRNA pool and, potentially, allow incorporation of alternative or mischarged tRNAs, thereby enabling synthesis of full‐length, although imperfect, OXPHOS polypeptides that can still contribute to respiratory‐chain assembly [91]. Importantly, deletion of only one *Clpp* allele was sufficient to improve mitochondrial protein synthesis, suggesting that partial inhibition may provide benefit without reproducing the consequences of complete CLPP deficiency [91]. These findings raise the possibility that other disorders caused by defects in mitochondrial aminoacyl‐tRNA synthetases or selected translation factors could similarly benefit from slowing mitochondrial translation and reducing abortive protein synthesis.

A complementary mechanism emerged from the identification of CLPXP as a regulator of respiratory‐chain turnover. CLPXP targets several OXPHOS proteins, including subunits of CI, CII and CIII, and selectively controls the turnover of the matrix‐exposed N‐module of CI [9, 14]. Under physiological conditions, this salvage pathway removes damaged N‐module components and allows their replacement within pre‐existing CI, thereby maintaining respiratory competence without requiring degradation and resynthesis of the entire complex [9]. When CLPP is absent, N‐module subunits are stabilized and accumulate in free subcomplexes. Although this impairs CI quality control in otherwise healthy mitochondria, it can preserve respiratory‐chain material when CI assembly or stability is already compromised [9].

The benefit of restraining CLPXP‐mediated turnover was demonstrated beyond DARS2 deficiency. In mtDNA mutator cells, depletion of CLPXP stabilized CI peripheral‐arm subunits and fully assembled CI and improved cell proliferation [9]. Preservation of CI was also observed in cells unable to assemble CIII because of cytochrome b deficiency and in COX10‐deficient cells lacking functional complex IV (CIV). CLPP or CLPX depletion did not correct the primary CIII or CIV defects, but it limited the secondary loss of CI that accompanies disruption of respiratory supercomplexes [9]. The same principle extended to an organismal model: reducing *clpp‐1* expression in *Caenorhabditis elegans* carrying the CI mutation *gas‐1(fc21)* partially restored CI abundance and improved development and reproductive fitness, despite limited recovery of respiration or ATP production [9]. These results indicate that preserving residual OXPHOS architecture can improve cellular and organismal fitness even when catalytic activity is not fully restored.

The therapeutic relevance of this concept was further supported in mouse models of DARS2‐driven mitochondrial encephalopathy. *Clpp* deletion delayed the degeneration of Purkinje cells, cortical neurons, and hippocampal CA1 neurons, reduced neuroinflammation, and improved motor performance [16]. Unlike in the heart, CLPP loss did not substantially alter mitochondrial translation in the brain, highlighting the tissue specificity of the underlying adaptation. Instead, protection correlated with stabilization of CI and respiratory supercomplexes, preservation of mitochondrial ultrastructure, and improved mitochondrial redox balance [16].

Together, these studies identify CLPP as a context‐dependent modifier of mitochondrial dysfunction. Its loss is not generally protective and can be detrimental under basal conditions. However, in disorders characterized by defective mitochondrial translation specifically biallelic pathogenic variants in nuclear‐encoded mitochondrial aminoacyl‐tRNA synthetase genes (mt‐aaRS genes) [93], CI instability or secondary respiratory‐chain disassembly, reducing CLPP activity may slow the degradation of residual OXPHOS components and preserve mitochondrial function. The strongest experimental support comes from DARS2 deficiency, mtDNA mutator models and settings in which primary defects in CIII or CIV lead to secondary destabilization of CI [9, 16, 91].

These findings suggest a broader framework for identifying mitochondrial diseases that might benefit from CLPP inhibition. Candidate disorders may include defects in mitochondrial translation, including selected mitochondrial aminoacyl‐tRNA synthetase or mitoribosomal disorders, as well as diseases in which CI is intrinsically unstable or becomes secondarily destabilized by defects elsewhere in the respiratory chain. In such settings, reducing CLPXP‐mediated turnover could preserve OXPHOS components and respiratory‐chain architecture even without correcting the primary genetic defect. Rather than the affected gene alone, a more informative predictor of responsiveness may therefore be excessive turnover of residual CI components that can be stabilized by partial CLPP inhibition [9]. CII subunits have also been identified as CLPXP substrates [8], while several CV components emerged from proteomic screens [7] but remain functionally unvalidated. If confirmed as bona fide CLPXP substrates, their stabilization could further influence respiratory‐chain organization and, in the case of CV, potentially help preserve cristae architecture.

Conversely, CLPP inhibition is unlikely to benefit mitochondrial disorders uniformly. It would be expected to have limited value when stabilization of respiratory‐chain proteins cannot restore productive assembly or catalytic activity, or when mitochondrial dysfunction is dominated by severe mtDNA depletion, profound loss of mitochondrial mass, or irreversible defects in essential catalytic components. Disorders caused by CLPP deficiency itself would represent an obvious contraindication, while prolonged systemic inhibition could become detrimental by compromising mitochondrial protein quality control. Therapeutic application will therefore require partial, reversible, and tissue‐appropriate inhibition rather than complete suppression of CLPP activity.

More broadly, this concept may extend beyond inherited mitochondrial disease to acquired pathological conditions characterized by acute CI dysfunction or instability, including ischaemia‐reperfusion injury, as discussed below.

### 1.4 CLPP Modulation in Systemic Metabolism

Beyond its role in mitochondrial protein quality control, CLPXP regulates mitochondrial metabolism through selective turnover of respiratory‐chain components, translation factors and metabolic enzymes. Substrate screens have identified proteins involved in fatty‐acid oxidation, amino‐acid metabolism and pyruvate utilization, including several acyl‐CoA dehydrogenases, ornithine aminotransferase, pyruvate carboxylase and pyruvate dehydrogenase kinase isoforms [10, 14, 31]. CLPP loss therefore does not simply impair mitochondrial proteostasis but induces broad, tissue‐specific metabolic remodelling.

An unexpected consequence of this remodelling is protection from obesity and insulin resistance. Two independent studies showed that whole‐body CLPP‐deficient mice are lean, have markedly reduced adiposity and display improved glucose tolerance and insulin sensitivity under both normal chow and high‐fat feeding [10, 11]. Despite comparable or even increased food intake, CLPP‐deficient mice gained substantially less weight and were protected from diet‐induced hepatic steatosis [10, 11]. In white adipose tissue, CLPP loss was associated with smaller adipocytes, increased mitochondrial content and respiration, induction of PGC‐1α and other markers of mitochondrial biogenesis, and enhanced oxidative capacity. Improved glucose homeostasis was accompanied by increased peripheral glucose uptake and preservation of insulin responsiveness during high‐fat feeding [10, 11].

These effects, however, were not reproduced by selective loss of CLPP in liver or skeletal muscle, indicating that the favorable phenotype does not arise from a single major metabolic organ [10]. Instead, whole‐body CLPP deficiency appears to establish a coordinated systemic state involving altered substrate utilization, endocrine signalling and inter‐organ communication [10]. This distinction is important therapeutically as targeting CLPP in one tissue may not reproduce the metabolic benefits of whole‐body loss and could instead expose tissue‐specific liabilities.

The most prominent liability is impaired brown‐adipose‐tissue function. Whole‐body CLPP‐deficient mice cannot maintain body temperature during acute cold exposure because adaptive thermogenesis is severely compromised [10]. CLPP loss causes a pale, lipid‐rich BAT phenotype with enlarged lipid droplets, respiratory‐chain remodelling and reduced CI activity. Adipose‐ and brown‐adipocyte‐specific loss of CLPP reproduces this whitening phenotype, demonstrating that it is cell autonomous rather than secondary to systemic changes [94].

Unexpectedly, BAT whitening is not explained simply by reduced oxygen consumption or defective fatty‐acid oxidation. CLPP‐deficient brown adipocytes accumulate D‐2‐hydroxyglutarate, generated through reprogramming of serine metabolism. D‐2HG promotes lipid‐droplet expansion, alters chromatin‐associated gene expression and decreases nuclear stiffness, thereby linking mitochondrial dysfunction to epigenetic and mechanical remodelling of the nucleus [94]. Pharmacological inhibition of D‐2HG production or increased D‐2HG clearance reverses major aspects of this phenotype, identifying metabolite signalling rather than energetic failure alone as a driver of BAT dysfunction [94].

The opposing responses of white and brown adipose tissue illustrate the complexity of CLPP‐directed metabolic interventions. In white fat, CLPP loss increases mitochondrial abundance and oxidative capacity and contributes to resistance against nutrient excess. In brown fat, the same perturbation promotes lipid accumulation, nuclear remodelling and loss of thermogenic competence [10, 11, 94]. Thus, the systemic protection against metabolic syndrome cannot be interpreted as uniformly improved mitochondrial function; rather, it reflects a redistribution of metabolic activity and tissue‐specific adaptation to chronic mitochondrial stress.

Conversely, CLPP activity may be reduced in some models of metabolic liver disease, and restoration or activation of CLPP has been reported to ameliorate steatohepatitis‐associated pathology [95]. This further emphasizes that both the direction and timing of CLPP modulation are likely to be disease‐ and tissue‐dependent. Inhibition may be advantageous in obesity and insulin resistance, whereas preservation or activation of CLPP could be beneficial in settings where loss of mitochondrial proteostasis contributes directly to tissue injury.

Together, these studies establish CLPP as a powerful regulator of systemic metabolism but also expose the limitations of treating it as a uniformly beneficial target. Therapeutic development will require partial and reversible modulation, careful selection of the target tissue and disease stage, and biomarkers that distinguish beneficial metabolic adaptation from loss of tissue function. Rather than complete CLPP ablation, controlled modulation of CLPXP activity may offer a means to improve glucose homeostasis and resistance to nutrient overload while avoiding impaired thermogenesis and other consequences of chronic mitochondrial dysfunction.

### 1.5 Future Directions and Therapeutic Challenges

Looking ahead, the greatest therapeutic potential of CLPP may lie in transient, tissue‐specific and directionally tailored modulation rather than sustained systemic inhibition or activation. The central challenge will be to define when reduced CLPP activity is protective, when increased activity is beneficial, and how far CLPP can be modulated without compromising essential mitochondrial protein quality control.

During reperfusion, rapid oxidation of ischaemia‐accumulated succinate can drive CI‐dependent reverse electron transport and a burst of mitochondrial ROS. Whether transient CLPP inhibition can therapeutically interfere with this process remains to be established. Our preliminary findings in renal ischaemia‐reperfusion injury indicate that CLPP deficiency reduces succinate‐driven mitochondrial ROS production and limits oxidative and tissue damage, providing initial support for this hypothesis. These observations raise the possibility that transient reduction of CLPP activity could be beneficial in acute settings characterized by pathological CI‐dependent ROS generation, but dedicated pharmacological studies will be required to define whether this can be translated into a therapeutic strategy.

One potential future application is ischaemia‐reperfusion injury. During reperfusion, rapid oxidation of ischaemia‐accumulated succinate can drive CI‐dependent reverse electron transport and a burst of mitochondrial ROS. Whether transient CLPP inhibition can therapeutically interfere with this process remains to be established. However, preliminary results from our ongoing studies in renal ischaemia‐reperfusion injury indicate that CLPP deficiency reduces succinate‐driven mitochondrial ROS production and limits oxidative and tissue damage. These findings provide initial support for the hypothesis that reduced CLPP activity may be beneficial in acute pathological settings characterized by excessive CI‐dependent ROS production. Pharmacological studies will be required to determine whether transient CLPP inhibition can reproduce this protection and to define the appropriate timing and therapeutic window. Such an approach could be particularly relevant for predictable ischaemic insults, including kidney transplantation, cardiac surgery or planned vascular interventions.

More broadly, the potential benefits of CLPP inhibition may extend to conditions in which respiratory‐chain components become unstable or are subject to excessive turnover. The strongest evidence currently concerns CI, but CLPXP‐dependent degradation has also been described for CII subunits, while several CV components have emerged from proteomic substrate screens. Although the latter remain functionally unvalidated, their stabilization could potentially influence ATP synthase organization and, consequently, cristae architecture. Future work should therefore determine whether excessive turnover of specific respiratory‐chain components can serve as a molecular signature identifying conditions that may benefit from partial CLPP inhibition.

Conversely, pharmacological CLPP activation may be advantageous when selective destruction of mitochondrial fitness is desired, most clearly in cancer, or potentially in other settings in which accelerated removal of maladaptive mitochondrial proteins is beneficial. Yet excessive proteolysis can rapidly compromise respiratory capacity, metabolic flexibility, and tissue function. The effects of CLPP modulation are therefore unlikely to be predicted simply by disease category. Tissue identity, respiratory‐chain stability, metabolic state, and the capacity to adapt to mitochondrial stress are likely to determine whether inhibition or activation is beneficial.

Several mechanistic questions remain unresolved. The principles governing physiological substrate selection by CLPXP are still incompletely understood, as are the determinants that make some cells highly sensitive to CLPP perturbation while others tolerate substantial changes in protease activity. Defining the relevant substrate landscape in individual tissues and disease states will be important both for predicting therapeutic response and for identifying mechanisms of resistance. The polypharmacological nature of several currently available CLPP modulators further makes rigorous demonstration of target engagement essential.

An important and underappreciated consideration is that CLPX also operates independently of CLPP to regulate 5‐aminolevulinate synthase (ALAS), the rate‐limiting enzyme of haem biosynthesis [96, 97]. Perturbing CLPP may therefore indirectly alter CLPX abundance or availability, with consequences for haem metabolism and erythropoiesis that are distinct from CLPXP‐dependent proteolysis. Monitoring CLPX levels and haem‐pathway intermediates may consequently become important when evaluating the safety of CLPP‐directed therapies.

Progress toward clinical translation will require selective and reversible modulators, controlled dosing schedules, organ‐targeted delivery and biomarkers that report respiratory‐chain stability, redox state, proteotoxic stress and substrate accumulation. Rather than asking simply whether CLPP can be targeted, the key question is when, where and in which direction its activity should be manipulated. Defining these parameters will determine whether CLPP can be exploited to preserve mitochondrial resilience in vulnerable tissues or, conversely, to selectively dismantle mitochondrial fitness in disease.

## Funding

Research reported in this publication was supported by funding to A. T. from the Deutsche Forschungsgemeinschaft (DFG, German Research Foundation) SFB1218 (Project No. 269925409) and TR1018/12‐1. L.I. was funded through a DFG‐funded CCRC Graduate Program (GRK 2407).

## Conflicts of Interest

The authors declare no conflicts of interest.

## Data Availability

Data sharing not applicable to this article as no datasets were generated or analysed during the current study.
